# Immunohistochemical Assessment of Maspin, β-Catenin, and MMP-14 in Oral Potentially Malignant Lesions and Oral Squamous Cell Carcinoma: A Retrospective Observational Study

**DOI:** 10.3390/medicina61061037

**Published:** 2025-06-04

**Authors:** Oana Mihaela Condurache Hrițcu, Delia Gabriela Ciobanu Apostol, Ștefan Vasile Toader, Carmen Solcan, Daciana Elena Brănișteanu, Mihaela Paula Toader, Victor-Vlad Costan

**Affiliations:** 1 Department of Surgicals, Faculty of Dental Medicine, “Grigore T. Popa” University of Medicine and Pharmacy, 700115 Iasi, Romania; oana.condurache-hritcu@umfiasi.ro (O.M.C.H.); victor.costan@umfiasi.ro (V.-V.C.); 2 Department of Morpho-Functional Sciences I, Faculty of Medicine, “Grigore T. Popa” University of Medicine and Pharmacy, 700115 Iasi, Romania; delia.ciobanu@umfiasi.ro; 3 Department of Physiopathology, Faculty of Medicine, “Grigore T. Popa” University of Medicine and Pharmacy, 700115 Iasi, Romania; stefan.toader@umfiasi.ro; 4 Department Preclinics, Faculty of Veterinary Medicine, Iasi University of Life Sciences Ion Ionescu de la Brad, 8, Mihail Sadoveanu Alley, 700489 Iasi, Romania; carmensolcan@yahoo.com; 5 Department of Dermatology, Faculty of Medicine, “Grigore T. Popa” University of Medicine and Pharmacy, 700115 Iasi, Romania; daciana.branisteanu@umfiasi.ro

**Keywords:** oral cancer, oral potentially malignant disorders, Maspin, β-catenin, MMP-14, immunohistochemical analysis, tumor progression, biomarkers, inflammatory microenvironment

## Abstract

*Background and Objectives*: Oral cancer remains a critical global health burden. Oral potentially malignant disorders (OMPDs) such as leukoplakia and oral lichen planus can precede oral squamous cell carcinoma (OSCC). Inflammation, tissue remodeling, and dysregulated signaling pathways are central to malignant transformation. This observational study aimed to evaluate the expression patterns of Maspin, β-catenin, and MMP-14 by immunohistochemistry (IHC) in oral leukoplakia, oral lichen planus, OSCC, and normal mucosa, exploring associations with lesion type, with no prognostic inferences drawn from a single timepoint. *Materials and Methods*: Biopsy specimens from 67 patients presenting with oral lesions (27 leukoplakia, 22 lichen planus, 18 OSCC), and 10 healthy controls were collected between January 2015 and January 2023. Inclusion criteria were age over 18 years and no other chronic illness, and a histopathologic diagnosis of oral leukoplakia, oral lichen planus or OSCC. Exclusion criteria were smokers, alcohol abuse, and prior head and neck radiotherapy, prior immunosuppressive therapy, systemic inflammatory diseases, absence of histopathological confirmation of the clinical diagnosis, and squamous cell carcinoma of the vermilion. Two pathologists independently scored staining in 10 high-power fields. Normal mucosa served as baseline. Immunohistochemical analysis was conducted using specific antibodies targeting Maspin, β-catenin, and MMP-14. Marker expression was assessed using a semi-quantitative scoring system based on staining intensity and classified into four categories: negative (−), weakly positive (+) for 1–10%, moderately positive (++) for 11–50%, and highly positive (+++) for more than 50%. *Results*: Maspin showed moderate (++) cytoplasmic/nuclear staining in leukoplakia and lichen planus in 78% of cases and high (+++) in OSCC and stroma in all cases. β-catenin shifted from membranous moderate positivity in 100% of OPMD cases to cytoplasmic/nuclear high positivity in all cases of OSCC. MMP-14 showed positivity (+) in 89% of OPMDs and high positivity (+++) in 100% of OSCC. *Conclusions*: Maspin, β-catenin, and MMP-14 exhibit distinct expression patterns across lesion types. While Maspin may reflect early tissue remodeling, β-catenin and MMP-14 changes suggest Wnt signaling activation and matrix remodeling in OSCC. Longitudinal studies are needed to establish their predictive value. This observational study refrains from prognostic claims and instead highlights biomarkers for future validation.

## 1. Introduction

Despite modest gains in diagnosis and therapy, 5-year survival for oral squamous cell carcinoma remains just 64.4% in high-income settings—dropping to 43.7% for Stage IV disease [1]—underscoring an urgent need for molecular biomarkers that can predict malignant transformation, refine diagnostic accuracy, and guide personalized management [2]. Immunohistochemistry, by directly visualizing protein-level alterations within tissue architecture, is ideally suited to this task [3]. Candidate markers include Maspin—a serpin that modulates cell migration and apoptosis [4]; β-catenin—a key regulator of cell–cell adhesion and Wnt signaling whose nuclear accumulation denotes oncogenic activation [5]; and MMP-14—an enzyme driving extracellular matrix degradation and invasion, with stromal overexpression linked to aggressive OSCC behavior [6]. Although these markers have each been studied individually, evidence remains limited. Few investigations have incorporated normal mucosa controls or leveraged quantitative digital analysis, and none have assessed all three markers together in OPMDs versus OSCC [7,8,9]. Despite growing interest in molecular biomarkers, immunohistochemical data in OPMDs remain scarce, underscoring the need for comprehensive studies like ours [10]. We therefore aimed to characterize and compare the immunohistochemical expression patterns of Maspin, β-catenin, and MMP-14 across normal mucosa, common OPMDs, and OSCC in a Romanian cohort.

Oral leukoplakia (OLK), oral lichen planus (OLP), and actinic cheilitis (AC) each carry a quantifiable risk of malignant transformation—6.64% for OLK [10,11], 1.43% for OLP [12], and 14% for AC [13]—underscoring the urgent need for robust molecular biomarkers. Such biomarkers must first serve as predictors, identifying high-risk lesions before histological progression; second, as diagnostic adjuncts, refining and confirming lesion classification beyond routine microscopy; and third, as prognostic indicators, guiding surveillance intensity and therapeutic choices [14]. Immunohistochemical markers like Maspin, β-catenin, and MMP-14 offer these capabilities by revealing specific molecular alterations linked to malignant potential, thus enabling truly personalized management of OPMDs [3,15,16,17]. Nevertheless, many conventional tumor markers still lack the specificity required for reliable clinical application, highlighting the ongoing challenge of translating biomarker discovery into practice [8,18,19,20,21,22,23,24].

Recent research has increasingly focused on the immunohistochemical evaluation of Maspin, β-catenin, and MMP-14 to elucidate early events in oral malignant transformation [3,4,6].

Maspin, a non-inhibitory serpin, modulates cell migration, apoptosis, and proliferation, exhibiting heterogeneous cytoplasmic and nuclear localization in OPMDs and OSCC [4,25,26,27].

β-catenin, a key regulator of cell–cell adhesion and Wnt signaling, undergoes aberrant membranous-to-nuclear translocation in lesions such as leukoplakia and lichen planus, signaling oncogenic activation and correlating with disease progression [5,28].

MMP-14, a membrane-bound matrix metalloproteinase, drives extracellular matrix degradation and stromal invasion, with overexpression linked to aggressive OSCC behavior and poorer outcomes [6].

In this study, we compare the immunohistochemical expression patterns of these three markers across normal mucosa, OPMDs, and OSCC within a Romanian cohort to clarify their roles in malignant progression and identify novel targets for early intervention.

Few studies to date combine Maspin, β-catenin, and MMP-14 in OPMDs versus OSCC, and none in a Romanian cohort.

## 2. Materials and Methods

### 2.1. Study Design and Specimen Collection

This retrospective observational study was conducted using biopsy specimens collected from January 2015 to January 2023. A total of 77 patients aged between 25 and 65 years were included: 67 patients clinically and histopathologically diagnosed with one of the following conditions: oral leukoplakia (27 cases; Group 1), oral lichen planus (22 cases; Group 2), and oral squamous cell carcinoma (18 cases; Group 3), as well as 10 healthy controls (Table 1). Normal mucosa specimens were collected from healthy donors undergoing third-molar extractions to serve as baseline controls. Inclusion criteria were age over 18 years, no other chronic illness, and clinical and histopathological diagnosis of oral mucosal leukoplakia/oral mucosal lichen planus/oral mucosal squamous cell carcinoma. Exclusion criteria were smokers, alcohol abuse, prior head and neck radiotherapy, prior immunosuppressive therapy, systemic inflammatory diseases, absence of histopathological confirmation of the clinical diagnosis, and squamous cell carcinoma of the vermilion. Histopathological diagnoses were confirmed by the Departments of Anatomical Pathology at the “Sfântul Spiridon” Emergency Clinical Hospital and the “Grigore T. Popa” University of Medicine and Pharmacy in Iași. This observational study was approved by the “Grigore T. Popa” University Ethics Committee, Iași (No. 61/23.03.2021), and all participants provided written informed consent.

### 2.2. Reagents and Antibodies

The immunohistochemical evaluation of molecular markers was performed using the following antibodies: Maspin Antibody (SC-271694, Clone C-8), Anti-β-Catenin Antibody (SAB4500543-100UG, produced in rabbit via affinity isolation), and Anti-MMP-14 Antibody (SAB4501901-100UG, produced in rabbit via affinity isolation) and dilutions (Maspin 1:100, β-catenin 1:50, MMP-14 1:14). Maspin Antibody (SC-271694, Clone C-8) was purchased from Santa Cruz Biotechnology, Dallas, TX, USA; Anti-β-Catenin Antibody (SAB4500543-100UG, produced in rabbit via affinity isolation) and Anti-MMP-14 Antibody (SAB4501901-100UG, produced in rabbit via affinity isolation) were obtained from Sigma-Aldrich, St. Louis, MO, USA. The antibodies were used at the following dilutions: Maspin 1:100, β-catenin 1:50, and MMP-14 1:14. Sections were incubated overnight at 4 °C in a refrigerator and positive controls (breast and colon carcinoma) plus omission of primary antibody as a negative control.

Data for statistical analysis were extracted from the archives of the Anatomical Pathology and Prosecture Clinic of the “Sf. Spiridon” Emergency Clinical Hospital in Iași over a 4-year period, in compliance with current regulations.

### 2.3. Tissue Processing and Staining Protocols

Biopsy specimens were processed using standard paraffin-embedding techniques. A portion of each specimen was sectioned at a thickness of 4 µm and stained with hematoxylin-eosin (H&E) to confirm the histopathological diagnosis.

For immunohistochemistry (IHC), additional sections were deparaffinized in three successive xylene baths (1.5 min each) and rehydrated in three ethanol baths (1.5 min each). Antigen retrieval was performed in citrate buffer at 97–99 °C for 30 min. Endogenous peroxidase activity and nonspecific binding were blocked using Peroxidase and Protein Block solutions (10 min each). The sections were then incubated overnight at 4–6 °C with primary antibodies (Maspin, β-Catenin, and MMP-14). Following washes, the slides were incubated with a biotinylated secondary antibody for 10 min and with Streptavidin Peroxidase for another 10 min. Chromogenic development was achieved using DAB (30 µL DAB Chromogen in 1.5 mL substrate for 1–2 min), resulting in a brown precipitate at the antigen–antibody binding sites. Finally, the slides were counterstained with hematoxylin (1 min), dehydrated through sequential ethanol baths (1.5 min each), cleared in xylene (3 min), coverslipped, and examined under Olympus BX40 microscope (Olympus Corporation, Tokyo, Japan).

### 2.4. Evaluation of Immunohistochemical Staining

Immunoreactivity for Maspin, β-Catenin, and MMP-14 was benchmarked against tissue-infiltrating lymphocytes as an internal reference and classified on a four-tier scale: Negative (−), Weak Positive (+, 0–10% of cells), Moderate Positive (++), and Strong Positive (+++, intensity comparable to lymphocytes). Two blinded pathologists independently scored at least 1000 epithelial cells per case—across 10 high-power fields (40×). Staining location was recorded by compartment (Maspin: cytoplasmic and nuclear; β-Catenin: membranous, cytoplasmic, nuclear; MMP-14: membranous and cytoplasmic), noting whether patterns were focal or diffuse. In OPMDs, intensity was assessed uniformly along the full epithelial thickness with attention to basal and suprabasal layers; in OSCC specimens, scoring emphasized the invasive tumor–stroma interface and leading edge of tumor nests to capture marker activity at sites of active invasion.

For site-specific evaluation, OPMD sections were scored separately in the superficial and basal epithelial layers, with representative areas chosen to include any dysplastic foci; in OSCC specimens, analysis was focused on the invasive tumor–stroma interface and leading edge of tumor nests.

Normal mucosa served as baseline. All immunohistochemical slides were independently scored by two experienced pathologists using the semi-quantitative scale, after checking 10 high-power fields (40× magnification) for each slide. Discrepancies greater than 10% were resolved by joint review and consensus. No digital image-analysis software was employed.

### 2.5. Statistical Analysis

Data were compiled in an Excel database and analyzed using standard statistical software, (SPSS Inc., Chicago, IL, USA, version 26.0 for Windows), with a significance threshold set at *p* < 0.05. The Student’s *t*-test was used to compare means between groups (based on degrees of freedom), while the Chi-squared (χ^2^) test—with Yates’ correction when necessary—was employed to compare frequency distributions. Additionally, Odds Ratios (OR) and Relative Risks (RR) were calculated to compare the likelihood of disease between exposed and unexposed groups.

## 3. Results

### 3.1. Maspin Expression in Premalignant Oral Lesions

Maspin expression was evaluated in well-characterized OPMDs with known malignant potential—specifically homogeneous leukoplakia and erosive lichen planus, defined histopathologically in our cohort—alongside oral squamous cell carcinoma (OSCC). Only homogeneous leukoplakia (flat, uniformly white; WHO criteria) and erosive lichen planus (erosive–atrophic clinical type, confirmed by band-like lymphocytic infiltrate at the dermo-epidermal junction) were included as OPMDs, owing to their higher documented risk of malignant transformation. Nuclear and cytoplasmic immunoreactivity (IR) was evaluated and classified as negative (−), weak positive (+), moderate positive (++), or strong positive (+++).

In homogeneous leukoplakia, 21 cases showed strong cytoplasmic and nuclear Maspin expression (++), while six cases demonstrated moderate immunoreactivity (+) in both compartments. In erosive lichen planus, intense cytoplasmic expression (+++) combined with weak nuclear positivity (+) was observed in 18 cases, whereas four cases exhibited moderate cytoplasmic staining (++) and weak nuclear reactivity (+).

**Figure 1:**
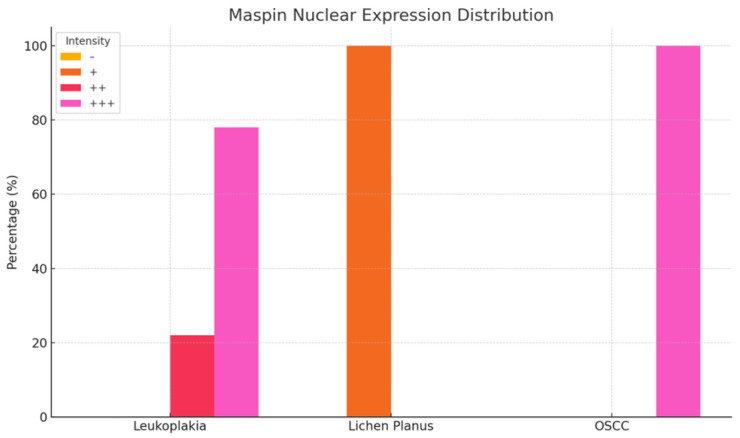
Nuclear immunoreactivity of Maspin in patients with leukoplakia, lichen planus, and squamous cell carcinoma.

**Figure 2:**
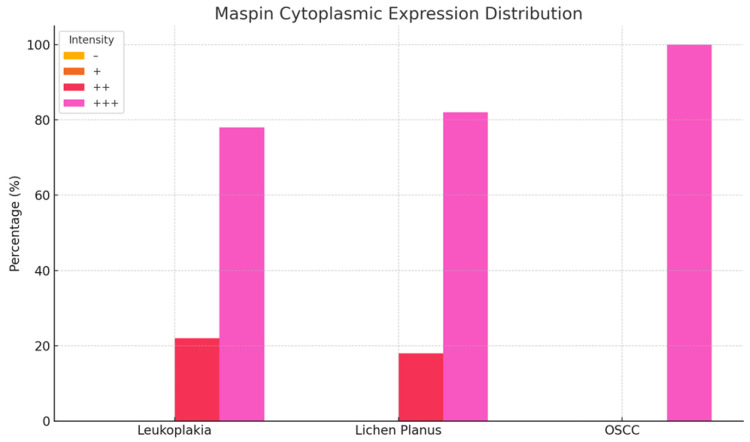
Cytoplasmic immunoreactivity of Maspin in patients with leukoplakia, lichen planus, and squamous cell carcinoma.

All 18 OSCC cases displayed strong Maspin expression (+++) in both the cytoplasm and nucleus (Figure 1 and Figure 2; Table 2).

**Figure 3:**
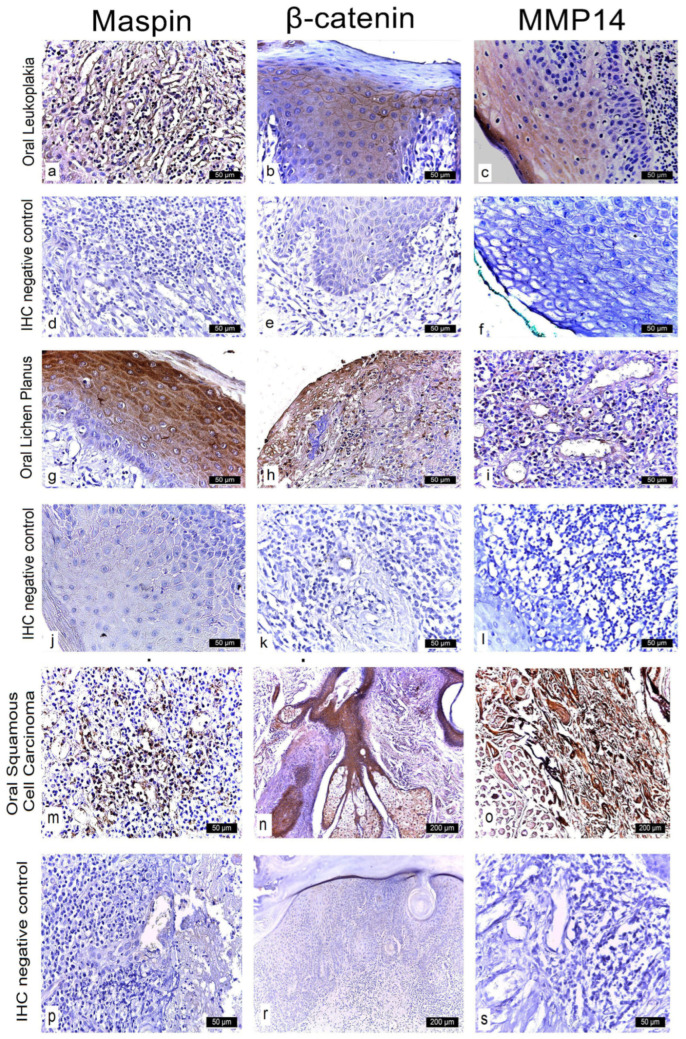
Immunohistochemical analysis. (**a**) Leukoplakia—jugal mucosa, ×40: section of stratified squamous epithelium with well-developed deep layers and granular cells overlying a loose, edematous dermis containing mononuclear cells, fine connective fibers, and neo vessels; focal, strong cytoplasmic and nuclear staining in the leukoplakic epithelium with moderate positivity in the underlying layers; (**b**) Leukoplakia—jugal mucosa detail, ×40 (ID 221345): stratified squamous epithelium with hyperkeratosis and incomplete keratinization, intracellular glycogen accumulation, occasional pyknotic nuclei in the stratum corneum, and homogeneous, intense cytoplasmic and membranous staining in the granular layer; (**c**) Leukoplakia—jugal mucosa, ×40: fragment of leukoplakic epithelium overlying loose papillary dermis with scattered inflammatory cells; moderate to weak, predominantly cytoplasmic staining in the lesional area; (**d**–**f**) IHC negative control ×40; (**g**) Lichen planus—jugal mucosa, ×40: stratified squamous epithelium with hyperkeratosis and underlying loose stroma containing connective fibers, granulocytic inflammation, isolated lymphocytes, and patent neo vessels; intense cytoplasmic and occasional nuclear positivity; (**h**) Lichen planus—buccal mucosa, ×40: stratified squamous epithelium with superficial erosions, hyperkeratosis, and loss of cohesion in basal and granular layers, a dense inflammatory infiltrate at the dermo epidermal junction and in papillary/deep dermis; strong cytoplasmic and focal nuclear staining in granular, stratum lucidum, and superficial layers; (**i**) Lichen planus—jugal mucosa, ×40: hypercellular stroma of lymphocytes and plasma cells with numerous thin-walled vessels and fibrous connective material; weak endothelial, membranous, and cytoplasmic staining in plasma cells and lymphocytes; (**j**–**l**) IHC negative control; (**m**) Squamous cell carcinoma ×40: stratified squamous epithelium with marked atypia, tissue disorganization, cellular pleomorphism, and dense mononuclear inflammation infiltrating tumor nests; immunostaining less pronounced in tumor nests but markedly intense in subjacent connective stroma; (**n**) Squamous cell carcinoma ×40: mononuclear cells (sometimes perivascular), islands of squamous epithelial tumor nests with intense cytoplasmic staining in granular and stratum lucidum layers; (**o**) Squamous cell carcinoma, ×40: buccal tissue fragment with underlying dermis of connective fibers of various sizes, small glands, vessels, and sparse inflammatory cells; intense staining in vascular, connective tissue, stromal cells, and along basement membranes; (**p**–**s**) IHC negative control ×40.

In homogeneous leukoplakia lesions, Maspin staining was focal in both the cytoplasm and nucleus of the superficial epithelium, while the deeper epithelial layers exhibited a moderate intensity—demonstrating a gradient of Maspin activity across different compartments (Figure 3a).

In erosive lichen planus, immunostaining revealed intense cytoplasmic positivity and focal nuclear expression, reflecting a pronounced inflammatory response and a potential immunomodulatory role for Maspin (Figure 3g).

In OSCC neoplastic epithelial cells at the invasive front in the stroma exhibited strong cytoplasmic and nuclear staining. These tumor aggregates demonstrated loss of intercellular cohesion, architectural disorganization, and a prominent inflammatory infiltrate. Maspin was absent in normal epithelial cells but highly expressed in neoplastic cells and the surrounding stromal tissue (Figure 3m).

Overall, these findings suggest that Maspin may play a pivotal role in the modulation of the tumor microenvironment and the progression of oral potentially malignant disorders.

### 3.2. β-Catenin Expression in Oral Lesions

Examination of homogeneous leukoplakia lesions revealed strong membranous and cytoplasmic β-catenin immunoreactivity (++) in 19 cases. An additional eight cases exhibited moderate membranous and cytoplasmic positivity (++).

In erosive lichen planus, β-catenin expression was strongly positive (+++) in the cytoplasm and moderately positive (++) in the nucleus in 16 cases, while six cases demonstrated moderate (++), diffuse cytoplasmic and nuclear immunoreactivity.

**Figure 4:**
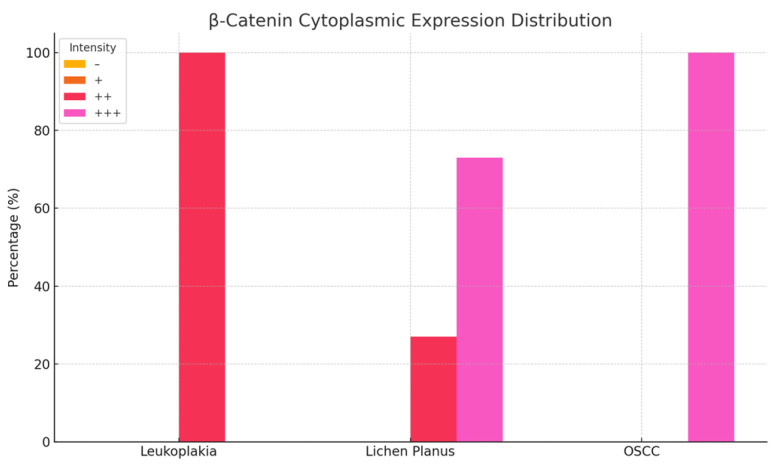
Nuclear immunoreactivity of β-catenin in patients with leukoplakia, lichen planus, and squamous cell carcinoma.

**Figure 5:**
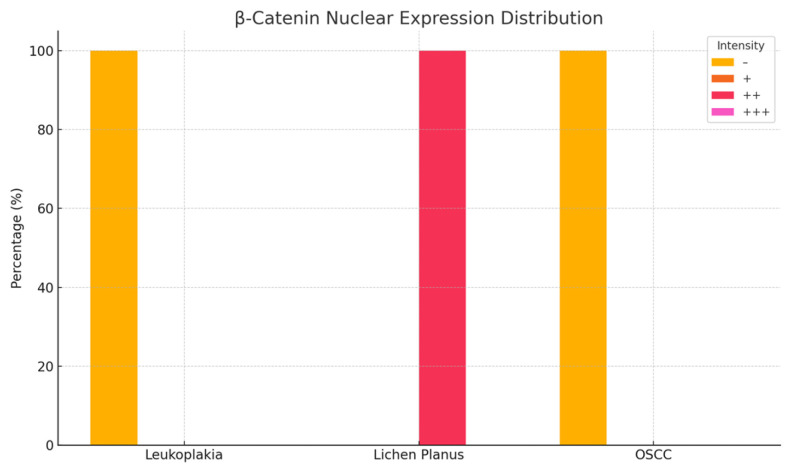
Cytoplasmic immunoreactivity of β-catenin in patients with leukoplakia, lichen planus, and squamous cell carcinoma.

In oral squamous cell carcinoma (OSCC), all 18 cases showed intense cytoplasmic β-catenin expression (+++) (Figure 4 and Figure 5; Table 2).

In homogeneous leukoplakia, pronounced β-catenin staining was also noted in the papillary dermis of the jugal mucosa, particularly within the elastic and muscular components of the vascular wall, suggesting a role in maintaining structural integrity and modulating localized inflammatory responses. The stratified squamous epithelium displayed hyperkeratosis, incomplete keratinization with intracellular glycogen accumulation, and pyknotic nuclei in the cornified layer. In the granular layer, β-catenin expression was uniformly intense in both the cytoplasm and membrane, indicating active cell proliferation and involvement in differentiation pathways (Figure 3b).

In erosive lichen planus, the epithelium exhibited superficial erosions, hyperkeratosis, and loss of intercellular cohesion in the deeper layers. Inflammatory infiltrates were present at the dermo-epidermal junction and within the papillary dermis (Figure 3h).

In OSCC, β-catenin exhibited strong cytoplasmic positivity, especially in stratum lucidum and the granular layer, indicating its potential role in tumor progression, epithelial reorganization, and disruption of tissue architecture (Figure 3n).

### 3.3. MMP-14 Expression in Oral Lesions

Immunohistochemical analysis of MMP-14 expression revealed variable staining patterns across different oral lesions. In homogeneous leukoplakia, 24 cases exhibited weak cytoplasmic immunoreactivity, while three cases were completely negative for MMP-14 expression in the membranous, cytoplasmic, and nuclear compartments.

**Figure 6:**
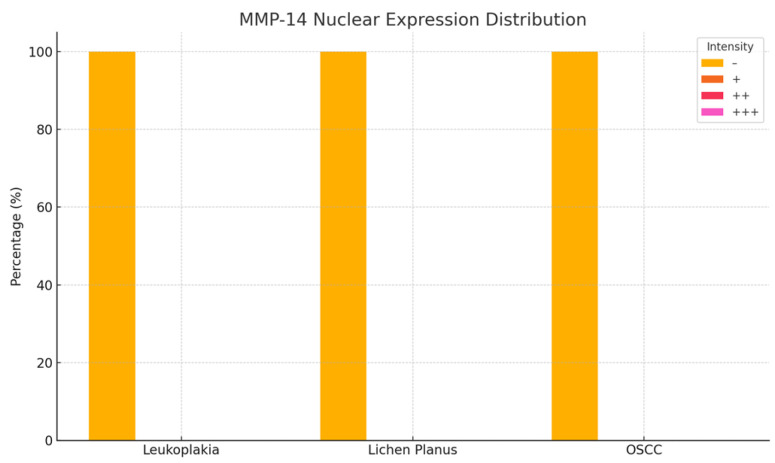
Nuclear immunoreactivity of MMP-14 in patients with leukoplakia, lichen planus, and squamous cell carcinoma.

**Figure 7:**
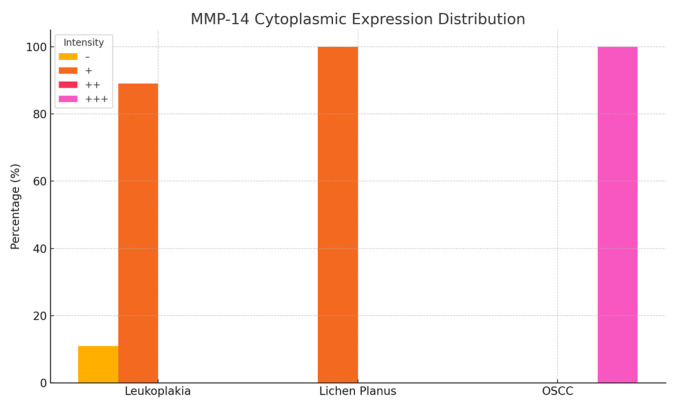
Cytoplasmic immunoreactivity of MMP-14 in patients with leukoplakia, lichen planus, and squamous cell carcinoma.

In erosive lichen planus, 18 cases demonstrated weak positive (+) staining localized to both the membrane and cytoplasm, whereas an additional four cases displayed weak cytoplasmic reactivity only. In contrast, all 18 cases of oral squamous cell carcinoma (OSCC) showed strong positive (+++) MMP-14 expression in both the cytoplasm and membrane (Figure 6 and Figure 7; Table 2).

Notably, leukoplakia lesions exhibited only moderate MMP-14 expression within the papillary dermis, suggesting a reduced level of extracellular matrix (ECM) remodeling relative to malignant lesions. These findings emphasize the critical role of MMP-14 in ECM degradation, tumor cell invasion, and angiogenesis, reinforcing its potential as a prognostic biomarker and a promising target for therapeutic intervention in oral lesions with malignant transformation potential (Figure 3c,i,o).

### 3.4. Comparison of Maspin, β-Catenin, and MMP-14 Expression

The three markers evaluated in our study—Maspin, β-Catenin, and MMP-14—exhibited distinct expression patterns that reflect their different roles in the pathogenesis of premalignant and malignant oral lesions.

Maspin shows moderate expression in leukoplakia and lichen planus, suggesting a role in maintaining tissue homeostasis. In squamous cell Maspin expression decreases, which may reflect the loss of an important regulatory mechanism in tumor invasion and malignant progression. Maspin is known for its function in inhibiting cell migration and invasion, and its reduced expression in malignant lesions may indicate an increased metastatic potential. Monitoring Maspin levels may be useful in assessing the risk of malignant progression.

β-catenin exhibits variable expression in premalignant lesions, with a predominantly cytoplasmic and membranous distribution. In lichen planus, expression is moderate to strong, supporting its role in cell adhesion and intracellular signaling. In squamous cell and epidermoid carcinomas, β-catenin expression becomes intense and is primarily localized in the cytoplasm and nucleus, suggesting increased oncogenic activity. This shift is associated with the activation of the Wnt signaling pathway, which is involved in cellular proliferation and invasion, indicating a higher malignant potential.

MMP-14 shows variable expression in lichen planus, and in potentially malignant lesions, this variability reflects extracellular matrix remodeling activity, essential for the initiation of malignant transformation. In squamous cell and epidermoid carcinomas, MMP-14 expression is significantly increased, highlighting its role in the degradation of extracellular matrix components, thereby facilitating tumor invasion and metastasis. Elevated levels of MMP-14 may correlate with tumor aggressiveness and serve as an important marker for identifying lesions with a high invasive potential.

These data highlight distinct biomarker expression patterns between OPMDs and OSCC, suggesting their utility for risk stratification.

## 4. Discussion

Our study provides valuable insights into the differential expression of Maspin, β-catenin, and MMP-14 in oral lesions with malignant transformation potential, highlighting their distinct roles in tumor suppression, cellular adhesion, and extracellular matrix remodeling.

Maspin, a serpin with tumor-suppressor properties, exhibited variable expression across different lesion types. Several studies have investigated Maspin’s role in oral lesions [29,30]. Xia et al. [31] reported that high Maspin expression is associated with reduced invasiveness in oral squamous cell carcinoma, supporting its tumor-suppressive function. Iezzi et al. [32] observed that decreased Maspin levels correlate with increased metastasis. Xia et al. [31] demonstrated that reduced Maspin expression in oral squamous cell carcinoma is linked to advanced tumor stage and poor prognosis. In addition, Iezzi et al. [32] found that lower Maspin levels in potentially malignant lesions such as oral leukoplakia and lichen planus were predictive of malignant transformation. Maspin expression is markedly upregulated in oral potentially malignant lesions, where it induces keratinocyte senescence and inhibits proliferation. Collectively, these findings indicate that Maspin functions as a tumor suppressor and underscores its prognostic value in oral cancer.

In homogeneous leukoplakia, our data demonstrated that most cases showed intense cytoplasmic and nuclear positivity for Maspin, suggesting robust tumor-suppressive activity in these early lesions. In contrast, erosive lichen planus displayed a spectrum of Maspin expression, with some cases demonstrating strong positivity and others moderate staining, reflecting a more complex role in modulating both cell growth and the inflammatory response. In oral squamous cell carcinoma, consistently high expression of Maspin was observed, indicating that its role may shift from tumor suppression to involvement in tumor–microenvironment interactions. High Maspin in OSCC stroma likely reflects reactive stromal remodeling rather than preserved tumor-suppressive function. The variable expression patterns of Maspin suggest that it could serve as a biomarker for both diagnostic and prognostic purposes and may inform therapeutic strategies that aim to modulate the tumor microenvironment.

β-Catenin is a multifunctional protein critical for cell–cell adhesion and Wnt signaling. Its aberrant expression—characterized by loss of membranous localization and nuclear translocation—has been implicated in oral carcinogenesis. Lequerica-Fernández et al. [33] demonstrated that altered β-catenin localization is closely linked to an unfavorable tumor immune microenvironment in oral cancer, with nuclear accumulation correlating with poorer outcomes. Similarly, Ramos-García and González-Moles [34] reported that loss of membranous β-catenin is significantly associated with increased tumor size, lymph node metastasis, and reduced survival in OSCC. Paluszczak [28] further emphasized that dysregulation of canonical Wnt/β-catenin signaling promotes oncogenic processes, including enhanced proliferation and migration.

Our observation of nuclear β-catenin aligns with Lequerica-Fernández et al. and Ramos-García et al., who associated this pattern with poor outcomes.

In addition, Reyes et al. [35] observed that increased nuclear β-catenin expression is significantly associated with the severity of epithelial dysplasia in oral potentially malignant lesions, suggesting a role in early malignant transformation. Complementing these findings, a recent narrative review by Yim and Laronde [36] highlighted that β-catenin, as a marker of epithelial-mesenchymal transition (EMT), undergoes significant expression changes during the progression from normal tissue through dysplasia to oral squamous cell carcinoma. Their work underscores the potential utility of β-catenin as an objective biomarker for risk assessment and prognostication in oral lesions. Collectively, these studies support the prognostic value of β-catenin in oral cancer. Aberrant β-catenin expression not only reflects disrupted cell adhesion but also signifies active oncogenic signaling, making it a promising target for therapeutic intervention and a valuable biomarker for stratifying patient risk.

MMP-14 is a membrane-bound metalloproteinase essential for extracellular matrix degradation and remodeling, which in turn facilitates tumor invasion and metastasis. In our study, MMP-14 expression was relatively weak in premalignant lesions and markedly elevated in oral squamous cell carcinoma (OSCC). This finding is in line with Noda et al. [6], who demonstrated that high-risk MMP-14 expression—particularly at the tumor–stromal interface and in metastatic lymph nodes—is strongly associated with extranodal extension and poorer prognosis in OSCC patients. Such evidence suggests that elevated MMP-14 levels in oral potentially malignant disorders may signal an early shift toward a more invasive phenotype. Moreover, increased MMP-14 expression has been linked to enhanced angiogenesis and a pro-metastatic microenvironment, underscoring its potential as both a prognostic biomarker and a therapeutic target. Together, these observations highlight the critical role of MMP-14 in oral cancer progression and support further exploration of strategies to modulate its activity for improved patient outcomes.

Our study reveals distinct expression patterns for Maspin, β-catenin, and MMP-14 in oral lesions with malignant transformation potential. Maspin, known for its tumor-suppressive properties, was strongly expressed in homogeneous leukoplakia with prominent cytoplasmic and nuclear staining, suggesting a protective role in early lesions. Maspin high expression in oral squamous cell carcinoma was likely due to increased stromal remodeling. β-Catenin, a key mediator of cell–cell adhesion and the Wnt signaling pathway, exhibited a shift from predominantly membranous localization in potentially malignant lesions to significant nuclear accumulation in more advanced dysplasia and carcinoma. This nuclear translocation implies activation of oncogenic transcriptional programs and disruption of cell adhesion mechanisms, contributing to tumor proliferation and invasion. MMP-14, a membrane-bound metalloproteinase essential for extracellular matrix degradation and remodeling, was expressed at low levels in potentially malignant lesions but was markedly upregulated in OSCC. Its overexpression facilitates tumor invasion, angiogenesis, and metastasis, indicating that elevated MMP-14 levels may serve as an early indicator of malignant transformation.

Collectively, these findings suggest that while high Maspin expression in early lesions may confer a tumor-suppressive effect, the aberrant nuclear localization of β-catenin and the upregulation of MMP-14 are associated with malignant progression. The combined evaluation of these biomarkers provides valuable diagnostic and prognostic insights and may guide the development of targeted therapeutic strategies to improve patient outcomes in oral cancer.

Our study is the first to integrate quantitative digital immunohistochemistry of Maspin, β-Catenin, and MMP-14 across well-characterized OPMDs and OSCC in a Romanian cohort, with rigorous scoring by two independent observers and inclusion of normal mucosa controls. However, it is limited by its single-center design, modest sample size (*n* = 67), absence of longitudinal follow-up data to correlate marker expression with clinical outcomes, and the inherent semi-quantitative nature of IHC despite digital validation. Future research should involve larger, multicenter cohorts to enhance generalizability, apply receiver-operating characteristic (ROC) analyses to define optimal biomarker thresholds, and incorporate prospective follow-up to validate the prognostic and predictive value of these markers.

## 5. Conclusions

Our results reveal distinct expression patterns of MMP-14, Maspin, and β-catenin across oral potentially malignant disorders and squamous cell carcinoma, reflecting their roles in matrix remodeling, tissue homeostasis, and Wnt-driven oncogenesis. MMP-14 was low-to-moderate in OPMDs but markedly elevated at the invasive fronts of carcinomas, consistent with its function in tumor invasion. Maspin showed moderate cytoplasmic and nuclear staining in leukoplakia and lichen planus but declined in carcinomas, suggesting loss of its metastasis-suppressive role. β-Catenin localized primarily to the membrane and cytoplasm in premalignant lesions but shifted to intense cytoplasmic/nuclear staining in OSCC, indicating Wnt pathway activation. Together, these biomarkers hold promise for risk stratification and may inform the development of targeted interventions in oral cancer.

## Figures and Tables

**Table 1:** Study Groups.

Group	Diagnosis	Number of Cases
Group 1	Oral Leukoplakia (OLK)	27
Group 2	Oral Lichen Planus (OLP)	22
Group 3	Oral Squamous Cell Carcinoma (OSCC)	18
Group 4	Normal mucosa	10
Total		77

**Table 2:** Immunohistochemical Expression of Maspin, β-Catenin, and MMP-14 in Oral Leukoplakia, Lichen Planus, and Squamous Cell Carcinoma.

Marker	Localization	Oral Leukoplakia (n = 27)				Oral Lichen Planus(n = 22)				Oral SCC (n = 18)			
		(−) n(%)	(+) n(%)	(++) n(%)	(+++) n(%)	(−) n(%)	(+) n(%)	(++) n(%)	(+++) n(%)	(−) n(%)	(+) n(%)	(++) n(%)	(+++) n(%)
Maspin	Nuclear	0(0%)	0(0%)	6(22%)	21(78%)	0(0%)	22(100%)	0(0%)	0(0%)	0(0%)	0(0%)	0(0%)	18(100%)
	Cytoplasmic	0(0%)	0(0%)	6(22%)	21(78%)	0(0%)	0(0%)	4(18%)	18(82%)	0(0%)	0(0%)	0(0%)	18(100%)
β-Catenin	Nuclear	27(100%)	0(0%)	0(0%)	0(0%)	0(0%)	0(0%)	22(100%)	0(0%)	0(0%)	0(0%)	0(0%)	0(0%)
	Cytoplasmic	0(0%)	0(0%)	27(100%)	0(0%)	0(0%)	0(0%)	6(27%)	16 (73%)	18(100%)	0(0%)	0(0%)	18(100%)
MMP14	Nuclear	27(100%)	0(0%)	0(0%)	0(0%)	22(100%)	0(0%)	0(0%)	0(0%)	0(0%)	0(0%)	0(0%)	0(0%)
	Cytoplasmic	3(11%)	24(89%)	0(0%)	0(0%)	0(0%)	22(100%)	0(0%)	0(0%)	18(100%)	0(0%)	0(0%)	18(100%)

## Data Availability

The data that support the findings of this study are available upon request from the corresponding author.
